# Redox Mechanism of Reactive Oxygen Species in Exercise

**DOI:** 10.3389/fphys.2016.00486

**Published:** 2016-11-07

**Authors:** Feng He, Juan Li, Zewen Liu, Chia-Chen Chuang, Wenge Yang, Li Zuo

**Affiliations:** ^1^Department of Kinesiology, California State University-ChicoChico, CA, USA; ^2^Department of Physical Education, Anhui UniversityAnhui, China; ^3^Affiliated Ezhou Central Hospital at Medical School of Wuhan UniversityHubei, China; ^4^Radiologic Sciences and Respiratory Therapy Division, School of Health and Rehabilitation Sciences, The Ohio State University College of MedicineColumbus, OH, USA; ^5^Interdisciplinary Biophysics Graduate Program, The Ohio State UniversityColumbus, OH, USA; ^6^Department of Physical Education, China University of GeosciencesBeijing, China

**Keywords:** dietary antioxidant, exercise, exercise-induced adaptation, ROS, skeletal muscle

## Abstract

It is well known that regular exercise can benefit health by enhancing antioxidant defenses in the body. However, unaccustomed and/or exhaustive exercise can generate excessive reactive oxygen species (ROS), leading to oxidative stress-related tissue damages and impaired muscle contractility. ROS are produced in both aerobic and anaerobic exercise. Mitochondria, NADPH oxidases and xanthine oxidases have all been identified as potential contributors to ROS production, yet the exact redox mechanisms underlying exercise-induced oxidative stress remain elusive. Interestingly, moderate exposure to ROS is necessary to induce body's adaptive responses such as the activation of antioxidant defense mechanisms. Dietary antioxidant manipulation can also reduce ROS levels and muscle fatigue, as well as enhance exercise recovery. To elucidate the complex role of ROS in exercise, this review updates on new findings of ROS origins within skeletal muscles associated with various types of exercises such as endurance, sprint and mountain climbing. In addition, we will examine the corresponding antioxidant defense systems as well as dietary manipulation against damages caused by ROS.

## Introduction

Regular exercise is beneficial to our health. However, unaccustomed or exhaustive exercise can result in detrimental health effects such as muscle damage, inflammation and oxidative stress. Specifically, repetitive muscle contraction involves accumulation of reactive oxygen species (ROS) (Zuo et al., 2011a, 2014, 2015b). These oxygen-derived free radicals or reactive derivatives, including superoxide (${\text{O}}_{2}^{•-}$), hydroxyl radical (•OH), and hydrogen peroxide (H_2_O_2_), have been implicated in various diseases and physiological conditions (Alfadda and Sallam, 2012). Acting as signaling molecules, a physiological level of ROS is essential for normal cellular functions. For instance, exogenous antioxidant supplements have been shown to suppress muscle contractility while the addition of H_2_O_2_ relieves such an effect, suggesting that oxidants (at low levels) may be imperative in facilitating muscle contraction (Reid et al., 1993; Powers and Jackson, 2008). However, the overproduction of ROS induced by exhaustive exercise training or other stresses, along with compromised antioxidant defenses, can lead to oxidative stress and related tissue damage (Powers et al., 2011b; Zuo et al., 2012). Interestingly, proper exercise (moderate to high intensity exercise) stimulates the adaptive responses and strengthens the endogenous antioxidant defense systems to combat excessive ROS thereby maintaining muscle redox balance (Parker et al., 2014; Zuo et al., 2015b).

Several techniques have been reported to examine oxidative stress in muscle tissues of both human and animal models (Powers and Jackson, 2008; Cheng et al., 2016). It is worth noting that the direct and quantitative measurement of ROS production continues to remain challenging in muscle redox biology due to the reactive nature of ROS as well as the methodological shortcomings. Commonly used indicators of ROS alteration in intact muscle fibers, such as fluorescent probes and spin traps, have limited specificity to the types of ROS (Powers and Jackson, 2008; Cheng et al., 2016). It is also difficult to assess the subtle changes in ROS levels during repeated muscle contractions directly using fluorescence (Cheng et al., 2016). Other indirect evaluation of oxidative stress includes the measurement of antioxidants, reduced/oxidized glutathione (GSH/GSSH) ratio, and oxidative modified molecules such as malondialdehyde for lipid peroxidation and 8-hydroxy-2′-deoxyguanosine for DNA oxidation (Powers and Jackson, 2008; Cakir-Atabek et al., 2010). These approaches are likely subject to experimental artifacts (Powers and Jackson, 2008). Along with limitations of these techniques on the accuracy of ROS measurement, the variation in specific ROS sources and oxidative modifications in different exercise protocols further contribute to the inconsistency and difficulty seen in this type of study.

Currently, the exact redox mechanisms underlying exercise-induced oxidative stress and exercise-induced adaptation remain unclear. Exploring ROS pathways may advance our understanding of muscle fatigue and recovery in exercise, as well as the development of potential tools for ROS assessment in exercising muscles. Although, mounting evidence has shown an elevation of oxidative stress associated with exercise, there is a lack of systemic review on how the activities of exercise (i.e., exercise type, intensity, and duration) affect ROS production. Therefore, this review aims to provide a timely update on the sources of ROS in different types of exercise, as well as the paradoxical role of ROS in acute and chronic exercise.

## ROS sources in muscle

Muscle activity has been shown to associate with ROS production, yet the extents and sources of ROS differ based on types of exercise (Steinbacher and Eckl, 2015). There is a general consensus that ROS are generated predominantly by contracting skeletal muscles during physical activity. Indeed, moderate levels of ROS are necessary for the production of normal muscle force; however, excess ROS can lead to muscle fatigue and contractile dysfunction (Powers et al., 2011a). Major endogenous sources of ROS in skeletal muscle include mitochondria, NADPH oxidase (NOX), and xanthine oxidase (XO) (Steinbacher and Eckl, 2015). Under physiological conditions, ROS are released as byproducts of cellular respiration by mitochondria. Accordingly, mitochondria-derived ${\text{O}}_{2}^{•-}$ can be observed in both resting and exercising muscle (Sakellariou et al., 2014; Zuo et al., 2015b). Mitochondrial respiration is in state 4 (basal) at rest, and enters active state 3 when muscle contraction begins, which is characterized by an increase in mitochondrial ADP levels due to rapid breakdown of ATP. Interestingly, the rate of ${\text{O}}_{2}^{•-}$ production is normally higher at basal mitochondrial respiration (state 4) than state 3 in both skeletal muscle and the diaphragm, suggesting that mitochondria might not be the major source of ROS in exercising muscles (Powers and Jackson, 2008; Kavazis et al., 2009; Sakellariou et al., 2014). On the other hand, NOX is a key ROS generator during muscle contractions, contributing to a larger extent of cytosolic ${\text{O}}_{2}^{•-}$ than mitochondria (Powers et al., 2011a; Steinbacher and Eckl, 2015). NOX is a multi-component enzyme located on the plasma membrane of phagocytic cells and several subcellular sites of skeletal muscle fibers (e.g., T-tubules and sarcolemma) (Michaelson et al., 2010; Zuo et al., 2011b; Sakellariou et al., 2013, 2014). NOX-induced ROS in the T-tubules can directly activate ryanodine receptor type 1 to enhance calcium (Ca^2+^) release and muscle contractions during exercise (Espinosa et al., 2006; Hidalgo et al., 2006). Other factors, such as phospholipase A_2_(PLA_2_), have been shown to stimulate NOX to produce ROS. PLA_2_ also facilitates phospholipid turnover and releases arachidonic acid (a substrate for lipoxygenases), leading to further ROS formation and lipid peroxidation related damage (Zuo et al., 2004; Steinbacher and Eckl, 2015). Found in the endothelium and cytosol of muscle, XO contributes to the production of extracellular ${\text{O}}_{2}^{•-}$ during isometric contraction. This XO-derived ${\text{O}}_{2}^{•-}$ plays a critical role in the muscle force generation (Powers and Jackson, 2008; Gomez-Cabrera et al., 2010). Moreover, the auto-oxidation of myoglobin or the oxidation of hemoglobin to methemoglobin further contributes to oxidative stress in the muscle by inducing peroxide formation (Marciniak et al., 2009).

In addition to endogenous sources of ROS described above, strenuous exercise-induced muscle injuries involve oxidative burst from immune cells, leading to a rapid ROS formation and subsequent oxidative damage (Steinbacher and Eckl, 2015). Particularly, untrained individuals are more prone to the detrimental effects exerted by the enhanced oxidative stress, while the trained subjects normally experience lessened effects due to increased oxidative tolerance (Steinbacher and Eckl, 2015). Aging or pathophysiological states of muscle are also associated with ROS elevation and contractile dysfunction (Steinbacher and Eckl, 2015). For example, greater endogenous oxidant generation has been observed in the isolated skeletal muscle fiber of old mice compared to young mice at rest (Palomero et al., 2013; Vasilaki and Jackson, 2013). It is suggested that such changes in ROS levels can be attributed to chronic inactivity of the muscle, which provides a possible explanation for the age-related ROS overproduction in muscle (Talbert et al., 2013; Vasilaki and Jackson, 2013). In addition, under disease states such as muscle dystrophy, simple stretch contractions can lead to significant muscle damage that is associated with ROS generation, through both increased NOX activation and cytosolic Ca^2+^ levels (Whitehead et al., 2010).

## ROS generation in various types of exercise

In skeletal muscle, both enzymatic (e.g., glutathione peroxidase (GPx) and catalase) and non-enzymatic (e.g., GSH, uric acid, bilirubin, vitamin E, vitamin C, etc.) antioxidants function as a unified complex to scavenge ROS (Powers and Jackson, 2008). These intracellular antioxidants are normally located within cells, cytoplasm, and organelles (e.g., mitochondria) to protect muscle fibers from ROS-induced damage (Powers and Jackson, 2008; Powers et al., 2011a). However, excessive ROS formation can offset these protective mechanisms during intense and exhaustive exercise. In general, the intensity of aerobic exercise is represented by maximal oxygen uptake (%VO_2max_) and the intensity of anaerobic exercise is described by repetition maximum (% RM). The extents and sources of ROS production can be influenced by the intensity, type, and duration of exercise, in which details will be discussed in latter paragraphs.

### Aerobic exercise

**Figure 1:**
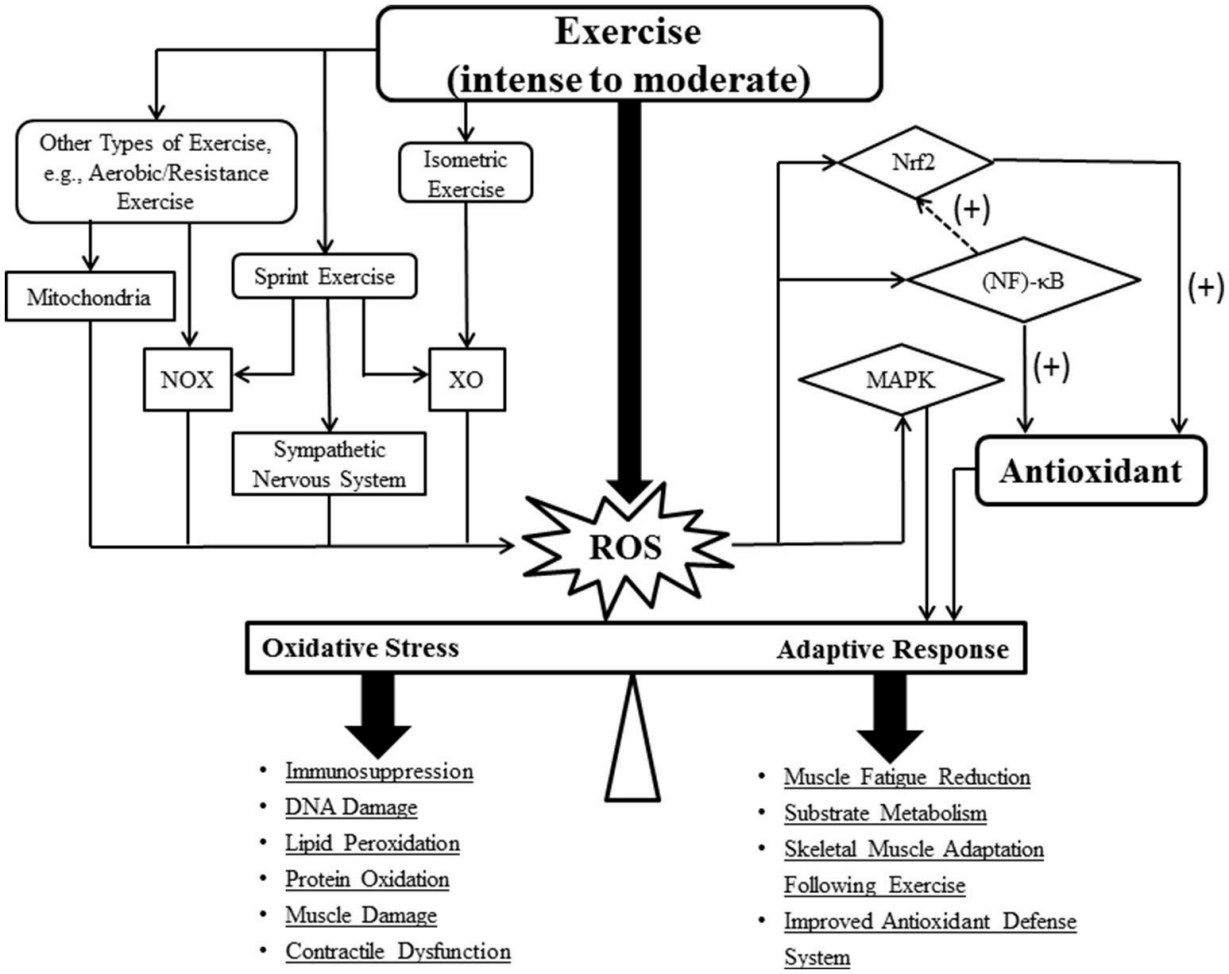
**Schematic illustrating ROS generation during different types of exercise and their associated roles in adaptive response**. The dash arrow represents an indirect effect. Abbreviations: reactive oxygen species (ROS); NADPH oxidase (NOX); xanthine oxidase (XO); mitogen-activated protein kinase (MAPK); nuclear erythroid 2 p45-related factor 2 (Nrf2); nuclear factor κB (NF-κB).

Strenuous aerobic or endurance exercise is commonly known to induce ROS and reactive nitrogen species overproduction due to enhanced metabolism, leading to oxidative stress and related injuries (Powers and Jackson, 2008; Neubauer et al., 2010; Gomes et al., 2012). It has been estimated that aerobic exercise results in a 1–3-folds increase of ${\text{O}}_{2}^{•-}$ during muscle contraction (Sakellariou et al., 2014; Figure 1). However, mitochondria only account for a small portion of ${\text{O}}_{2}^{•-}$ generation during aerobic exercise (Sakellariou et al., 2014; Zuo et al., 2015b). In fact, mitochondria-derived ${\text{O}}_{2}^{•-}$ formation in skeletal muscle is decreased during exercise compared to that at rest. This is because contractile activities alter the redox status in muscles toward a more oxidative state, leading to a lowered mitochondrial NADH/NAD^+^ ratio. The decline in NADH/NAD^+^ ratio is linked with reduced complex I-dependent ${\text{O}}_{2}^{•-}$ release (Sakellariou et al., 2014). During endurance exercise, ATP is broken down to release energy and support continuous muscle contraction. In some instances, AMP is formed which can be further degraded to hypoxanthine, xanthine, and uric acid through a biochemical process involving XO. As described previously, XO induces ${\text{O}}_{2}^{•-}$ formation by utilizing molecular oxygen, thereby exacerbating oxidative stress (Mastaloudis et al., 2001). Elevated lipid peroxidation and DNA oxidative damage have been observed following a single bout of intensive exercise. Such acute inflammatory and oxidative responses can be induced by vigorous aerobic exercise, which resemble the stress responses following ischemic stroke and myocardial infarction (Mastaloudis et al., 2004). In addition, oxidative burst induced by leukocytes is an effective mechanism for fighting against microbes during infection (Saran et al., 1999; Agarwal et al., 2003). The long-lasting endurance exercise may compromise the ROS-generation capability of leukocytes, resulting in an increased susceptibility to infectious diseases in athletes (Nielsen et al., 2004). Moreover, for exercising people with diseases such as asthma, special cautions must be taken since asthma may cause substantial ROS formation and oxidative stress thus compromising exercise-induced benefits (Jiang et al., 2014).

Although, single bouts of intensive aerobic exercise may cause potential oxidative damage to muscle fibers, regular aerobic exercise will help enhance the cellular ability to detoxify ROS over-accumulation (Radak et al., 2013). Regular/moderate exercise has been shown to enhance antioxidant defense by incrementing the activity of endogenous antioxidant enzymes such as superoxide dismutase (SOD), GPx, and catalase (Miyata et al., 2008). Exercise protects the body against constant mild or moderate ROS exposure through redox-associated preconditioning including oxidative damage repair systems (Radak et al., 2013). This moderate exercise-mediated adaptation also involves increased myocellular antioxidant capacity which helps to lower ROS levels (Mastaloudis et al., 2001; Knez et al., 2006). Moreover, increased ROS formation in active skeletal muscles plays a critical role in exercise adaptation by modulating muscle contraction (Mastaloudis et al., 2004; Radak et al., 2013). For example, endurance running is regarded as important for survival in human evolution since it can trigger exercise-associated adaptive responses through metabolic and redox challenges (Radak et al., 2013; Ferraro et al., 2014; Wiggs, 2015). However, contemporary lifestyles decrease physical activities and suppress human adaptive capacity of metabolism and redox homeostasis (Radak et al., 2013). Substantial evidence has suggested that at least 30 min of accumulated physical activity (moderate-intensity) each day is necessary to maintain good health and reduce potential disease risks (Knez et al., 2006). Accordingly, Berzosa et al. and Georgakouli et al. both observed a significant elevation of plasma total antioxidant capacity in healthy individuals after a 30 min of submaximal exercise (70% of maximum workload and 50–60% of the heart rate reserve, respectively) on cycle ergometer (Berzosa et al., 2011; Georgakouli et al., 2015).

### Anaerobic exercise

Although the main source of ROS during aerobic exercise has been thoroughly reviewed in a previous study (Powers and Jackson, 2008), little is known regarding the potential source of ROS during short-term intensive (anaerobic) exercise such as sprints. The redox mechanisms of anaerobic work have been investigated in a variety of exercise models including sprinting trainings as well as isometric and eccentric exercises (Nikolaidis et al., 2007, 2008; Stagos et al., 2015).

Unlike other exercises, sprints predominantly rely on anaerobic energy pathways due to its high energy demand. While sprinting, a small portion (0.15%) of ${\text{O}}_{2}^{•-}$ is produced in the mitochondria (St-Pierre et al., 2002). This lower than usual ROS production in skeletal muscle mitochondria can be attributed to relatively low amounts of oxygen consumption and increased ADP (state 3) during sprints (Herrero and Barja, 1997; Morales-Alamo and Calbet, 2014). NOX is one of the potential sites of ${\text{O}}_{2}^{•-}$ production associated with intense muscle contractions (Sakellariou et al., 2013; Figure 1). Additionally, XO activation triggered by an elevation in hypoxanthine during and following sprints, is regarded as another important contributor for ROS production (Kang et al., 2009; Figure 1). Intensive exercise accelerates ATP degradation, leading to elevated formation of AMP, hypoxanthine, xanthine, and uric acid. Particularly, the increased levels of xanthine facilitate ROS generation by XO, thereby exacerbating oxidative stress in anaerobic exercise (Mastaloudis et al., 2001; Radak et al., 2013). In response to intense exercise, the active sympathetic nervous system can also play a role in ROS formation (Figure 1). Accordingly, Bors et al. demonstrated that adrenaline administration largely increased H_2_O_2_ levels *in vitro* (Bors et al., 1978).

In static positions, isometric exercise is common in daily activities such as holding weighted objects. A variety of oxidative stress biomarkers have been examined in response to isometric exercise; yet mixed results can be produced. For instance, isometric contractions result in increased levels of hydroperoxide and elevations in blood protein carbonyls. However, there is no change in plasma malondialdehyde (a useful indicator of lipid peroxidation) (Rodriguez et al., 2003; Urso and Clarkson, 2003). Moreover, repetitive static exercise (RSE) can be considered as a similar condition to partial ischemia/reperfusion, which may protect the tissues against oxidative stress (Zuo et al., 2013). However, Sahlini et al. observed no signs of ROS elevation during prolonged RSE despite a manifestation of decreased mechanical efficiency and force generation (Sahlin et al., 1992). Furthermore, isometric exercise was reported to induce an increase in the GSSH/GSH ratio, but intense isometric contraction can lead to lactic acidosis and stimulate the conversion of ${\text{O}}_{2}^{•-}$ to highly reactive •OH (Waterfall et al., 1996; Groussard et al., 2000; Garatachea et al., 2012).

A handful studies have also assessed the oxidative stress resulting from eccentric exercise (Nikolaidis et al., 2007, 2008), a physical activity that can induce sarcolemma inflammation and subsequent ROS overproduction and muscular damage (Nikolaidis et al., 2007, 2008). One study reported that ROS formation peaked after the large muscle function decline in downhill running (Close et al., 2004). Other study showed that eccentric contraction likely causes secondary muscle damage due to ROS–induced inflammation (Nikolaidis et al., 2007; Silva et al., 2010).

### Mountain climbing

A good example for exploring the influence of ROS on physical activity is mountain climbing. Mountain climbing involves the exposure to extreme environmental conditions caused by high altitudes, stimulating ROS generation in the body (Miller et al., 2013). Mountaineers generally experience various undesirable conditions at altitudes of 2 km or above (Basnyat, 2001; Hackett and Roach, 2001; Basnyat et al., 2003). For example, long-term exposures to an altitude above 4 km could induce a loss of appetite, leading to nutrition deficiency and weight loss (Siesjö et al., 1996; Wasse et al., 2012). Collectively, these symptoms associated with acute mountain sickness are related to harsh environmental factors such as low oxygen, cold, and ultraviolet rays (Askew, 2002; Smedley and Grocott, 2013). Particularly, hypobaric hypoxia generates a large amount of ROS, resulting in the subsequent tissue injuries in mountaineers (Askew, 2002; Julian et al., 2014).

As altitude increases, lower atmospheric pressures lead to reduced atmospheric oxygen partial pressures and arterial blood oxygen levels, causing hypoxic damage (Askew, 2002; Vallecilla et al., 2014). Under normal circumstances, people are able to resist mild oxidative stress and restore redox balance via the body's naturally equipped antioxidant system. However, overwhelmed antioxidant defenses due to severe oxidative stress (e.g., inappropriate exercise exertion) can promote cell damage or death (Bakonyi and Radak, 2004; Zuo et al., 2015b). Oxidative stress induced by hypoxia at high altitudes results in intracellular Ca^2+^ overflow, energy metabolism disruption and cellular organelles oxidation (Askew, 2002; Mungai et al., 2011). It is noted that such damage can occur in both aerobic and anaerobic exercises at any exercise intensity under hypoxic conditions (Bakonyi and Radak, 2004).

Moreover, physical exercise associated with mountain climbing also plays an important role in ROS production (Askew, 2002), as physical workouts at high altitudes can aggravate oxidative stress (Bakonyi and Radak, 2004; Miller et al., 2013). For example, enhanced DNA breakage and oxidation were frequently observed in exercising subjects at high altitudes compared to sea level (Møller et al., 2001; Ziogas et al., 2010). The antioxidant system in the body is particularly vulnerable under stressed conditions such as hypoxia, and is unable to prevent DNA damage caused by exercise at high altitudes (Møller et al., 2001). In addition to physical exercise and hypobaric hypoxia, other environmental factors including coldness, sunburn and diet also contribute to the augmentation of oxidative stress at high altitudes (Askew, 2002). Insufficient antioxidant intake may exacerbate high altitude-induced anorexia as well as tissue damage (Askew, 2002; Bailey et al., 2004). Thus, caution should be taken at high altitudes as mountaineers could experience intense oxidative stress from both high altitude environments and physical workouts.

## ROS-induced adaptive response to exercise

In the past decades, majority of studies mainly emphasize on the detrimental effects of exercised-induced oxidative stress on muscles, whereas researchers recently reported the significance of ROS in triggering and mediating body's adaptive responses to exercise (Yavari et al., 2015). Acute exercise generates excessive ROS that cause damage in the body, while regular exercise results in bodily adaptations leading to resistance against oxidative damage via antioxidant pathways (Yavari et al., 2015). It has been observed that the antioxidant capacity of skeletal muscle can be altered by exercise training. For example, SOD levels are commonly higher in the resting blood and muscle of trained individuals compared to those of control groups (Tiidus et al., 1996). Endurance training may increase the activities of SOD and GPx in both plasma and exercised muscles (Lambertucci et al., 2007; Brooks et al., 2008; Vieira Junior et al., 2013; Azizbeigi et al., 2014). This magnitude of exercise-mediated changes in SOD or GPx activities is dependent on the intensity and duration of that specific exercise. For example, high-intensity exercises may lead to a higher muscular GPx activity than that in low-intensity ones (Powers et al., 1994; Fisher et al., 2011). Similarly, long-duration exercise trainings (e.g., 60 min/day) increase more muscular GPx function than short-duration (30 min/day) exercise bouts (Powers et al., 1994). The enhancement of exercise-induced SOD and GPx activity is fiber type-specific, and a greater increase is normally observed in skeletal muscles mainly composed of highly oxidative fibers (e.g., type I and type IIa) (Powers et al., 1994; Gonchar, 2005; Ferraro et al., 2014). However, whether catalase (another major antioxidant enzyme) expression or activity can be affected by chronic exercise remains controversial, as previous studies reported mixed results (Vincent et al., 2000; Brooks et al., 2008; Liberali et al., 2016).

Several important pathways have been proposed in mediating the adaptive responses to exercise training (Morris et al., 2008; Samjoo et al., 2013; Csala et al., 2015). It is suggested that mitochondrial ROS generated during regular exercise are necessary for the activation of primary signaling pathways associated with muscle adaptation (Yavari et al., 2015). Nuclear factor erythroid 2-related factor (Nrf2), a redox-sensing transcription factor, is the primary regulator of antioxidants as well as other cytoprotective cofactors that are responsible for the enhanced antioxidant defense system (Osburn and Kensler, 2008; Muthusamy et al., 2012). Upregulated Nrf2 expression occurs after high-intensity exercise (Gounder et al., 2012). In a mouse myocardium, acute exercise activates Nrf2 signaling via increased ROS production, which in turn, promotes the trans-activation of antioxidant genes, leading to improved cardioprotection (Muthusamy et al., 2012; Figure 1). However, there is a lack of human studies that address the Nrf2-mediated adaptive responses generated by exercise. Another adaptation to exercise involves the enhancement of mitochondrial biogenesis via upregulated peroxisome proliferator-activated receptor-γ coactivator-1α (PGC-1α) gene expression (Steinbacher and Eckl, 2015). PGC-1α has been demonstrated to upregulate Nrf2 in order to control mitochondrial biogenesis (Wu et al., 1999; Wright et al., 2007). The upstream signals that regulate PGC-1α expression such as mitogen-activated protein kinase (MAPK) and nuclear factor (NF)-κB are redox-sensitive (Dodd et al., 2010; Derbre et al., 2012). In addition, proteasome inhibition, which reduces NF-κB activation, has been shown to enhance cellular antioxidant defenses via an Nrf2-dependent transcriptional mechanism, suggesting the indirect effects of NF-κB on antioxidant regulation (Karin and Ben-Neriah, 2000; Elliott et al., 2003; Dreger et al., 2009; Figure 1). Exercise-induced ROS also plays a role in adaptation through the oxidation of cysteine residue in various proteins. For example, cysteine-rich peroxiredoxin, an antioxidant responsible for H_2_O_2_ catalysis, is oxidized and formed stable dimers in response to elevated H_2_O_2_ levels during exercise, managing H_2_O_2_ gradients and regulating extracellular redox-signaling (Wadley et al., 2016). Moreover, the disulfide bonds formed by oxidized cysteine residues likely enhance protein synthesis in active individuals (Buresh and Berg, 2015).

Exhaustive endurance and/or resistance exercise may induce temporary immunosuppression (i.e., a reduction in CD4/CD8) (Jin et al., 2015). Particularly, the elevated oxidative and physical stress reflected by the level of intracellular ROS and cortisol, respectively, may contribute to the immunosuppression (Jin et al., 2015; Figure 1). For example, NF-κB is activated in response to stimulants such as H_2_O_2_, TNF-α, and other proinflammatory cytokines (e.g., IL-6). The activated NF-κB then binds to a specific DNA binding domain and upregulates the corresponding antioxidant gene expression (e.g., SOD) (Morgan and Liu, 2011; Figure 1). Accordingly, the NF-κB signaling pathway can be activated following an acute bout of exercise in rats (Ji, 2007). In addition, low levels of inflammatory markers have been observed in the elderly who frequently exercise (Marzatico et al., 1997). As mentioned previously, MAPK also plays an important role in exercise-induced adaptation in skeletal muscle. MAPK is composed of four subfamilies (ERK1/2, JNK, p38 MAPK, and ERK5) (Kramer and Goodyear, 2007; Figure 1). The activities of ERK and MEK have a positive correlation with exercise intensity in human skeletal muscle (Widegren et al., 2000). ROS such as H_2_O_2_, can induce the activation of ERK, JNK, and p38 MAPK in skeletal myoblasts in a dose- and time-dependent manner (Kefaloyianni et al., 2006). Oxidative stress could also modulate the MAPK signaling pathway through insulin signaling and glucose transport (Kim et al., 2006; Sandström et al., 2006; Kramer and Goodyear, 2007; Figure 1).

## Antioxidant intervention

Growing evidence on exercise-induced oxidative damage and impaired muscle performance has prompted intensive research into the efficacy of antioxidant supplementation in exercising individuals (Gomes et al., 2012). It has been suggested that oral antioxidant supplements, which are common intakes among athletes, support endogenous antioxidant defense system against oxidative stress (Peternelj and Coombes, 2011). However, studies on the effects of antioxidant supplements in muscle damage prevention and recovery remain inconsistent, mostly due to different exercise protocols, research designs, and analytical methods (Peternelj and Coombes, 2011).

Most commonly known antioxidants are vitamins, which can be obtained readily through natural foods such as vegetables and fruits (Trapp et al., 2010). Indeed, vegetarians have been shown to have higher levels of endogenous vitamin than omnivores due to antioxidant–rich diets, providing effective protections against exercise-induced oxidative stress (Rauma and Mykkanen, 2000; Trapp et al., 2010). Similar nutritional strategy is wisely utilized by the athletes to improve performance and promote hastened muscle recovery (Margaritis and Rousseau, 2008). Antioxidant vitamins have demonstrated potential prophylactic effects. In the study performed by He et al., short-term combined vitamin C and E supplementation not only attenuated levels of creatine kinase (a muscle damage marker) and muscle soreness, but also enhanced muscle protection following the second bout of aerobic exercise (He et al., 2015). Moreover, a supplemental or adequate intake of nutritional antioxidants is necessary for endurance athletes (Wagner et al., 2010). For example, long-distance runners who took vitamin C and E for 4 or 5 weeks prior to a marathon experienced less muscle damage (Urso and Clarkson, 2003). Likewise, Fogarty et al. reported that both short- and long-term supplementation of watercress, which is rich in lipid soluble antioxidants (i.e., α-tocopherol, β-carotene, and xanthophyll), can reduce exhaustive exercise-associated lipid peroxidation and DNA damage (Fogarty et al., 2011). Phenolic compounds found in grapes also exhibited great antioxidant and anti-inflammatory properties, and has been shown to improve exercise performance in recreational runners (15% increase in time-to-exhaustion running) (Ali et al., 2010; Toscano et al., 2015). Moreover, Pala et al. suggested that coenzyme Q10 supplementation protects tissue from oxidative injury during exercise training through a mechanism involving Nrf2 expressions (Pala et al., 2016).

Despite beneficial effects mentioned above, a thorough understanding on the application of vitamin and antioxidant supplements such as effective dosage and administration method is necessary to avoid undesirable effects. Some studies have indicated that antioxidant supplements fail to protect against the damaging effects of oxidative stress such as exercise-induced lipid peroxidation and inflammation, both of which hinder muscle recovery (Teixeira et al., 2009). Specifically, prolonged antioxidant supplementation is not recommended since it can disrupt endogenous antioxidant levels and interfere exercise-induced adaptation, thereby blunting body's defense against oxidative stress (Peternelj and Coombes, 2011; Rowlands et al., 2012). Excessive antioxidant intake, such as vitamin C and E supplementation, has been shown to delay healing process and muscle strength restoration in athletes following an exhaustive exercise training (Margaritis and Rousseau, 2008; Theodorou et al., 2011). Additionally, an increased exercise-induced oxidative stress is observed in individual taking high-doses of α-tocopherol (Margaritis and Rousseau, 2008). In short term, N-acetyl-cysteine (NAC; antioxidant) and allopurinol (an inhibitor of XO) do attenuate muscle damage and lipid oxidation caused by acute exhaustive exercise (Gómez-Cabrera et al., 2003; Braakhuis and Hopkins, 2015). Nevertheless, long-term intakes of these antioxidants may not be beneficial (Braakhuis and Hopkins, 2015). Gomez-Cabrera et al. further suggested that 8 weeks of vitamin C supplementation prevents training-induced mitochondrial biogenesis by suppressing the expression of SOD and GPx (Gomez-Cabrera et al., 2008). A double-blinded and placebo-controlled study also showed that the combination of vitamins C and E blunts mitochondrial adaptive responses (i.e., increase in COX4 protein) after 11 weeks of endurance training (Paulsen et al., 2014).

Collectively, mixed results from antioxidant intervention studies may be interpreted by the variances in participants' baseline redox status, the dose and length of the antioxidant supplementation, and the choice of oxidative stress markers. Instead of antioxidant supplements, a balanced diet consisting natural antioxidants from fruits and vegetables is sufficient to meet the dietary requirement for physically active individuals (Bloomer et al., 2007; Poljsak et al., 2013; Yavari et al., 2015).

## Perspectives

In the past decades, exercise-induced oxidative stress and its effects have been largely studied. Despite the increasingly sophisticated approaches on the study of ROS in skeletal muscle, inconsistency in the results of several studies remains, which is likely associated with different methodology of ROS measurements and exercise protocols. It is therefore essential to determine an appropriate measuring module for various types of exercises and muscles in order to obtain reliable and valid data (Zuo et al., 2015a; Jackson, 2016). Excessive ROS production beyond the capability of antioxidant defense following exhaustive and/or unaccustomed exercise could adversely affect human adaptive responses. The current challenge is the lack of in-depth human studies that explore the molecular mechanisms of how ROS regulate the key redox-sensitive transcription factors including Nrf2, NF-κB, MAPK and PGC-1α. Further studies focusing on minimizing oxidative damage and maximizing adaptive response induced by exercise are indispensable. Developing promising strategies that combine an effective natural antioxidant diet with customized exercise within a variety of populations (e.g., disease population, obese individuals, the elderly, and trained/untrained individuals) could tremendously improve health and quality of life. Moreover, identifying the effective and reliable biomarkers of alterations in redox homeostasis is critical in monitoring the training tolerance of individuals and may shed a light on optimizing a customized training program.

## Author contributions

LZ, FH designed the outline, FH, JL, ZL, CC, WY, LZ wrote the paper.

### Conflict of interest statement

The authors declare that the research was conducted in the absence of any commercial or financial relationships that could be construed as a potential conflict of interest.
